# Quality of medicines in Sri Lanka: a retrospective review of safety alerts

**DOI:** 10.1186/s12913-023-09995-3

**Published:** 2023-09-12

**Authors:** Jegath Janani Tharmalinga Sharma, Madumai Ketharam, Kaumada Binoli Herath, Senathiraja Sherley Shobia

**Affiliations:** 1 Ministry of Health, Nutrition & Indigenous Medicine, Colombo South Teaching Hospital, Baddegama Wimalawansa Thero Mawatha, Colombo, Sri Lanka; 2 National Medicines Regulatory Authority, Colombo, Sri Lanka; 3 Institute for Research and Development in Health & Social Care, Colombo, Sri Lanka

**Keywords:** Quality, Medicines, Withhold, Recall, Safety alerts, Sri Lanka

## Abstract

**Background:**

Many medicine quality problems are detected after they arrive at health facilities. Thus, critically defective medicines that may pose health risks to patients need to be withheld or recalled.

**Aims:**

To investigate the withheld and recalled medicines in relation to the types of defects, their total numbers, therapeutic categories, pharmaceutical dosage forms, and country of manufacturer during the study period.

**Methods:**

A retrospective review was performed on withheld and recalled medicines published on the publicly available National Medicines Regulatory Authority (NMRA) official website in Sri Lanka between June 2018 and August 2021. Details on substandard medicines (SM) were extracted and documented. Each record of SM was individually reviewed to determine the type of defect, subsequent action taken by NMRA, therapeutic category, pharmaceutical dosage form, and country of manufacturer.

**Results:**

A total of 163 defects were identified in 143 defective medicines, among which the most common types of defects were contamination (*n* = 59, 36.2%), stability defects (*n* = 41, 25.2%), packaging and labelling defects (*n* = 27, 16.6%) and active pharmaceutical ingredient defects (*n* = 26, 15.9%). Out of 143 total defective medicines identified, anti-infectives accounted for 41.9%, while parenteral preparations (44.0%) were found to be frequently defective. Nearly 70% of the recalled and withheld medicines were of Indian origin, and some manufacturers were identified to be repeatedly involved.

**Conclusions:**

This study revealed that contamination was the most frequent cause of defective medicines, while parenteral preparations and anti-infectives were the most susceptible pharmaceutical dosage form and therapeutic category found to be substandard, respectively. In addition, the findings show that some manufacturers were accountable for repetitive withholdings and recalls, which reflects the ignorance of quality control measures and weak regulatory inspections as a violation of Good Manufacturing Practice (GMP).

**Supplementary Information:**

The online version contains supplementary material available at 10.1186/s12913-023-09995-3.

## Background

The importance of good quality, safety and efficacy of medicines for effective treatment is well known [1, 2]. The World Health Organization (WHO) states that “an estimated 1 in 10 medical products in low- and middle-income countries (LMICs) is substandard or falsified” [3]. A systematic review including fifteen studies in 25 different countries, mainly in Africa and Asia, showed that the median prevalence of substandard medicines was 28.5% (range 11% – 48%) [4]. Furthermore, published data have reported that the global prevalence of counterfeit medicines ranges from 1% – 50% [5, 6].

The literature frames poor-quality medicines as two kinds: ‘substandard medicines’ and ‘falsified or counterfeit medicines’. WHO defines ‘substandard medicines’ as (which are also called ‘out of specification’) “authorized medical products that fail to meet either their quality standards or their specifications, or both” and ‘falsified or counterfeit medicines’ as “medicines that are deliberately and fraudulently mislabelled with respect to identity composition or source” [7]. Substandard medicines and falsified medicines will be referred to as ‘SM’ and ‘FM’, respectively.

SM and FM are global medication safety issues and a major burden on public health [8–11]. Quality failures of medicines could result in patient harm. They have been linked to causing deaths worldwide [10–13], contributing to antimicrobial resistance [13, 14], poisoning, treatment failure, and adverse drug reactions [15–18]. In addition to causing patient harm, the use of SM and FM can have an enormous economic impact on individuals and healthcare providers [6, 10]. Eventually, these outcomes jeopardize the entire healthcare system.

Batches of medicines are sometimes recalled or withheld when quality, safety, efficacy issues or defects are detected [19, 20]. Such problems are usually detected when patients, consumers or healthcare providers report issues on medicines to relevant authorities [19, 20]. Medicine recalls have been reported all around the world, including the West [8, 9, 19, 21, 22]. SM and FM can be recalled due to several factors, and to provide accurate information, they can be classified into multiple defect categories, as reported in the literature [8, 9, 19, 21, 22]. Furthermore, the European Medicines Agency (EMA), an agency of the European Union in charge of the evaluation and supervision of pharmaceutical products, classifies SM and FM in five high-level terms. These five high-level terms include manufacturing laboratory control issues, product contamination and sterility issues, product label issues, product packaging issues, and product physical issues [23].

Thus, medicine recalls are sought through a set of specified standards. The National Medicines Regulatory Authority (NMRA), an independent authority governed by a board, is Sri Lanka's central regulatory body. This authority is responsible for ensuring that all medicines in the country consistently meet the established quality, safety and efficacy requirements [24–26]. NMRA uses active and passive surveillance methods to monitor the quality of medicines in the Sri Lankan market. Active surveillance involves post-marketing surveillance inspections of medicines, including visual inspection, labelling assessment and sampling of medicines analysed through the National Medicines Quality Assurance Laboratory (NMQAL). Through the passive surveillance method, NMRA receives alerts about defective, suspected SM and FM from other institutions, such as the WHO, hospitals, patients and the pharmaceutical industry. To fulfil it’s one of the objectives, the NMRA has recently launched a website to publish information regarding recalls and withholds of medicines since 2018 [27]. However, information such as the types of defects that caused recall or withhold, the therapeutic category and pharmaceutical dosage forms of medicines that were recalled or withheld have not been studied in Sri Lanka. Therefore, this study aimed (I) to characterize and categorize the types of defects that caused withhold or recall and their total numbers; (II) to investigate the total number of withholds and recalls of medicines with respect to their therapeutic categories and pharmaceutical dosage forms; and (III) to examine the country of the manufacturers.

## Methods

### Study design

A descriptive cross-sectional study was conducted using publicly available information on withheld and recalled medicines published between June 2018 and August 2021 (details of 25 months) on the NMRA official website (www.nmra.gov.lk).

### Sampling technique

A search for all medicines withholds, recalls, market withdrawals and safety alerts received and/or issued by NMRA was carried out by reviewing the publicly available database kept at NMRA headquarters. NMRA actively started publishing records on the website for medicines withholds and recalls alerts in 2018. To assess the documents, a search was performed for documents recorded between June 2018 and August 2021. All data available from the NMRA official website during the study period were purposively sampled.

### Inclusion criteria and exclusion criteria

All defective medicines for human use withheld or recalled due to quality-related issues or falsification published on the NMRA website from June 2018 to August 2021 were included in the study, while data on substandard medical devices, cosmetics, borderline products, and alerts such as FM that were not identified in Sri Lanka were excluded from this study.

### Study process

**Fig. 1:**
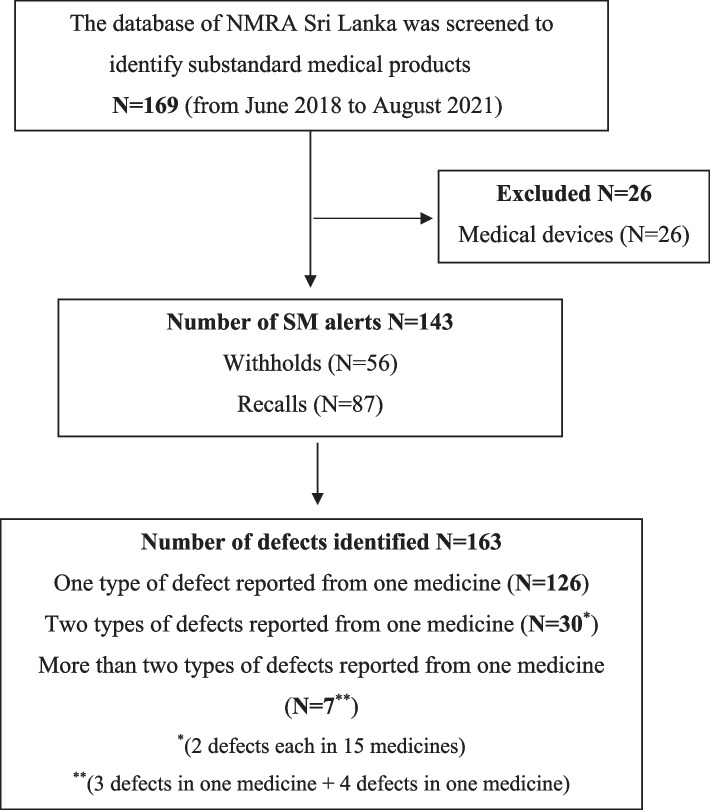
Flow chart depicting the search strategy

A structured Excel database was created to collect data, analyse and consolidate the medicine withholds and recalls. The records fulfilling the inclusion criteria were sequentially numbered, and the following information was extracted from the alerts: name of the medicine, strength, pharmaceutical dosage form, country of manufacturer, reported defect, and subsequent action by NMRA. Each record was reviewed to characterize the defects, determine the total number of cases with respect to therapeutic categories and pharmaceutical dosage forms and examine the country of manufacturer. Two categories of defective medicine alerts were identified: withholds and recalls of SM (Fig. 1).

#### I. Characterization and categorization of defects

Characterization of defects was based on the cause for withholds or recalls published on the NMRA website. The categorization was performed according to a modified version of a classification used in a similar study conducted in the UK and Canada by Almuzaini T et al., [8, 9]. Out of the six main defect categories presented by Almuzaini T et al., only four were included in the modified classification, namely: contamination; stability defects; active pharmaceutical ingredient (API) defects; packaging and labelling defects. Delivery defects and potency defects were replaced by three new categories: variation in physical properties (such as weight and volume); adverse events reported; and unclassified. The reported defects were reviewed according to WHO guidelines [28–30] and other reported literature [8, 9, 19, 21] by two reviewers (JJTS, MK) and were discussed with a third reviewer (KBH) for general agreement. Any disagreements were discussed until a consensus was reached. Descriptions of the seven main defect categories are given in Table 1.

**Table 1:** Descriptions of the seven main defect categories

Defect category	Defect description
I. Contamination	‘The undesired introduction of impurities of a chemical or microbiological nature, or of foreign matter, into or onto a starting material or intermediate during production, sampling, packaging or repackaging, storage or transport’ [28].
II. Stability defect	‘Stability is the ability of a pharmaceutical product to retain its chemical, physical, microbiological and biopharmaceutical properties within specified limits throughout its shelf-life’ [29]. Therefore, if this criterion was not met, the case was considered as a stability defect.
III. Active pharmaceutical ingredient (API) defect	‘Any substance or mixture of substances intended to be used in the manufacture of a pharmaceutical dosage form, to furnish pharmacological activity or other direct effect in the diagnosis, cure, mitigation, treatment, or prevention of disease or to affect the structure and function of the body’ [28]. API should be within the specified limits of the assay (pharmacopoeial standard). Therefore, if this criterion was not met, the case was considered as API defect.
IV. Packaging and labelling defects	‘Any material, including printed material, employed in the packaging of a pharmaceutical, but excluding any outer packaging used for transportation or shipment. Packaging materials are referred to as primary or secondary according to whether or not they are intended to be in direct contact with the product’ [28]. Defects related to labelling and packaging were included in this category.
V. Variation in physical properties such as weight and volume	Failure to comply with pharmacopoeial specifications stated under uniformity of weight or volume [30] were considered in this category.
VI. Adverse events reported	Cluster of adverse events reported were considered in this category.
VII. Unclassified	Cases which could not be included to any of the above categories were considered in this category.

#### II. Total number of withheld and recalled medicines with respect to their therapeutic categories (ATC) and pharmaceutical dosage forms

Although the WHO Anatomical Therapeutic Chemical (WHO/ATC) classification system classifies drugs according to five different levels [31], this study only focused on ATC level 1 categorization (which consists of 14 main anatomical or pharmacological groups) to classify medicines into their respective therapeutic categories. Each medicine was fed into the ATC/DDD Index website [31] to find the relevant WHO/ATC level 1 category.

Furthermore, the total number of withheld and recalled medicines in relation to the pharmaceutical dosage form of medicines was also assessed.

#### III. Country of manufacturer of withheld and recalled medicines

The country of manufacturer of those withheld or recalled medicines was also examined.

### Data analysis

Descriptive statistics were used to quantify the following data using the Microsoft Excel package: total number of withholds and recalls, total number of medicines that were withheld and recalled, number of withheld and recalled cases in relation to the types of defects, therapeutic categories, pharmaceutical dosage forms, and country of manufacturer during the period of study time. Proportions or cross-tabulations of frequency data were also conducted to determine the percentages of types of defects. However, to calculate the prevalence of quality failed medicines in Sri Lanka requires a denominator such as the total number of medicines available during the study period. Since this count is unknown, the prevalence could not be assessed.

## Results

### Trends in reported cases

Since NMRA started publishing defective medicine alerts online, a total of 169 SM alerts were identified from June 2018 to August 2021, of which 143 alerts (56 withholds and 87 recalls) complied with the aforementioned inclusion criteria. From these 143 defective medicines, the analysis showed a total of 163 defects, of which a single defect was reported from a majority (*N* = 126), while others reported two or more defects. A flow chart depicting the search strategy is given in Fig. 1.

Although reporting online defective medicine alerts started in 2018, only two cases had been published in the same year. The total numbers of withholds and recalls published in 2018, 2019, 2020, and 2021 were 2, 66, 36, and 39, respectively.

#### I. Characterization and categorization of defects

Out of 143 medicines analysed, a total of 163 defects were identified. The most common defect observed was contamination, which accounted for 59 (36.2%) cases including physical and microbiological contaminations. The second most frequent was stability defects (25.2%), which were reported mostly in tablets and capsules. Next in line were packaging and labelling defects (16.6%) and the defect of API being out of specification (15.9%). However, only one case was related to an ADR. Defects by categories, subcategories and total numbers reported are presented in Table 2.

**Table 2:** Defects by categories and subcategories

	**Main categories (n, %)**	**Subcategories**
I	Contamination (*n* = 59,36.2)	Colour variations, impurities, outside standards (*n* = 49)
		Lack of sterility (*n* = 7)
		Microbiological contamination (*n* = 3)
II	Stability defects (*n* = 41, 25.2)	Unspecified stability failure (*n* = 33) Failure in dissolution test (*n* = 8)
III	Active pharmaceutical ingredient (API) defects (*n* = 26, 15.9)	API out of specification (either more or less) (*n* = 26)
IV	Variation in physical properties such as weight and volume (*n* = 4, 2.4)	Weight variation (*n* = 3) Volume variation in single dose parenteral preparation (*n* = 1)
V	Packaging and labelling defects (*n* = 27, 16.6)	Failure in container closure system functionality (*n* = 6) Packaging in a wrong carton (*n* = 1) Manufacturer's information missing (*n* = 4) Contain a lesser number of tablets than stated (*n* = 1) Failure to comply with the description as per the manufacturer’s specifications (*n* = 7) Labelling errors (*n* = 8)
VI	Adverse events reported (*n* = 1, 0.6)	A cluster of adverse drug reaction (ADR) events reported (*n* = 1)
VII	Unclassified (*n* = 5, 3.0)	Data not adequate to assess (*n* = 5)

Total number of defects reported (*N* = 163)

A detailed table presenting the defects by categories and subcategories with examples is attached as Additional file 1. 

#### II. Withheld and recalled medicines with respect to their therapeutic categories (ATC) and pharmaceutical dosage forms

Out of 143 medicines analysed, the most frequently reported categories were anti-infectives (41.9%), medicines acting on the nervous system (18.8%) and medicines acting on the alimentary tract and metabolism (12.5%). Furthermore, very few cases were reported for therapeutic classes such as respiratory system preparations (02), blood and blood-forming organ preparations (02), dermatologicals (03) and musculoskeletal system preparations (01). The total number of withheld and recalled medicines with respect to their therapeutic categories are presented in the table shows this in more detail (see Additional file 2).

Eight types of pharmaceutical dosage forms were identified according to the type of formulation and route of administration. Parenteral preparations were the most susceptible dosage form, for which a notable number of defects (44.0%) were identified. Out of 63 cases related to parenteral preparations, 33 were anti-infectives. The second most reported pharmaceutical dosage form was tablets (34.2%). Pharmaceutical dosage forms of withheld and recalled medicines are presented in the table shows this in more detail (see Additional file 3).

#### III. Country of manufacturer of withheld and recalled medicines

**Fig. 2:**
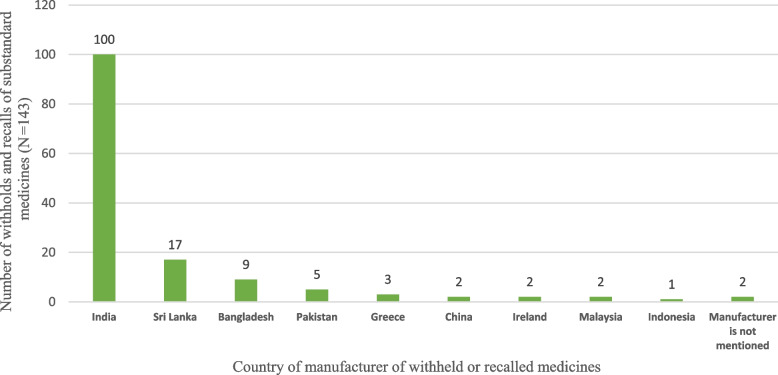
Total numbers of withheld and recalled medicines by country of origin

The country of manufacturer of each withheld and recalled medicine was examined. Nearly 70% of the recalled and withheld medicines were of Indian origin. Sri Lanka, Bangladesh and Pakistan accounted for 11.8%, 6.2% and 3.4% of defective medicines, respectively, while only a few were reported from other countries. However, the two products did not contain manufacturer details. Furthermore, seven manufacturers were identified as repeatedly involved with medicine and recalls. Figure 2 shows the numbers of withheld and recalled medicines by country of origin.

## Discussion

The present study results indicate a total of 163 defects in 143 defective medicines, and these 143 medicines had been recalled or withheld due to single or multiple defects. This result is consistent with findings from other studies conducted internationally examining medicine recalls. Different numbers of defective medicines, such as 653, 280, 195, 84, and 83, have been reported in Canada [9], the UK [8], the USA [19], Saudi Arabia [21] and Zambia [22], respectively. However, it should be noted that it is not possible to compare the compatibility of findings from this study with other international studies due to the difference in study duration. Despite the numbers reported, defective medicines remain a challenge and pose serious negative consequences. Although it is not possible to quantify the clinical outcomes precisely, they can prolong illness and inconvenience, cause drug resistance, and may result in financial losses.

In this study, the most frequently reported type of defect was contamination (36.2%). This finding is compatible with studies conducted by Almuzaini T et al. [8], and Alquadeib BT et al., [21]. Similarly, an analysis conducted to characterize drug recalls issued over 30 months by the Food and Drug Administration (FDA) in the USA reported that the most common reason for recall was contamination (50.1%) [32]. These results demonstrate that contamination continues to be challenging in the manufacturing process of medicines, which needs to be addressed promptly. Moreover, stability defects (25.2%) and API defects (15.9%) were also found in considerable numbers. However, in contrast, the Canadian study reported by Almuzani T et al. [9], revealed that the most frequent type of defect was stability issues (32%). These stability and API defects must be taken into account, as they have the potential to affect the bioavailability of the active ingredients in the systemic circulation and in turn, leading to therapeutic failure.

The consequences of using such medicines may vary from treatment failure to major adverse events and even death. In this study, the ADR observed in patients was identified as a consequence of reaching a defective medicine to patients. This medicine had been withheld due to a cluster of adverse events that occurred (severe headache) soon after the injection was injected (gentamycin sulphate BP) and was the only case found after it had reached patients. Furthermore, it was not able to find any information (from the NMRA website) about the other defective medicines, whether they had reached the patient or caused any harm. However, it is noteworthy to report these defects, as these circumstances also have the capacity to cause medication errors.

This present study identified parenteral preparations (44%) had been routinely reported as defective, which is consistent with the results of a study conducted in the UK [8]. There is a serious threat to patient safety with defective parenteral preparations as they enter systemic circulation directly. Additionally, anti-infectives (41.9%) were the most reported therapeutic group in this study. In the case of anti-infectives, the development of drug resistance is also a major threat. Kelesidis and colleagues have also documented that antibiotic and antiparasitic medicines appear far more likely to be substandard than other medicines [15]. Furthermore, they stated that antibiotics are more susceptible to degradation while transporting or storing at temperatures above 25 °C with high humidity; therefore, stability issues can occur in the tropical climate under real storage conditions (in warehouses or some wholesale pharmacies) [15]. Sri Lanka falls under climatic zone IVb, as per categorization by the WHO expert committee on specifications for pharmaceutical preparations, in this case, medicines are expected to be tested for stability at 30 °C/75% relative humidity levels [33]. Therefore, the storage condition must be tested accordingly and maintained throughout the shelf life to prevent deterioration.

Furthermore, this study revealed that most defective medicines were imported, and some foreign manufacturers were accountable for repetitive withholds and recalls. It was observed that nearly 70% of defective medicines were of Indian origin, and Gautam CS et al., reported that India is also a major exporter of counterfeit medicines to developing and least-developed countries [5]. This issue has significant economic consequences, which can directly impact the pharmaceutical budget of the country. SM that are not recalled are not only a threat to patient safety but also a huge economic burden to LMICs, such as Sri Lanka, especially providing free public healthcare services.

To our knowledge, this is the first review done on the issue of quality failed medicines in Sri Lanka by assessing the safety alerts reported on the NMRA website. In addition, this analysis was based on secondary data obtained from the NMRA official website (www.nmra.gov.lk/) [27] and this NMRA is the central regulatory body in Sri Lanka that ensures that the medicinal products available in the country meet applicable standards of safety, quality and efficacy. Therefore, this is the only and most credible source that could be found as an information source on medicinal product alerts. Thus, this study provides important insight into regulatory policy and practice. To avoid medicine quality failures, a favourable supply chain management plan would need to be set up by relevant authorities in Sri Lanka addressing reasons for medicine withholds and recalls. In addition, medicine defect reporting culture should be encouraged, barriers such as lack of awareness, overcomplicated reporting systems, and lack of feedback mechanism from regulatory authorities need to be addressed, and regulatory policies must be tightened. It is also recommended that manufacturers, importers, suppliers or market authorization holders should be held accountable for ensuring the quality of medicines through increased awareness, education, and sanctions.

However, we acknowledge that this study has several limitations. An in-depth analysis of these reported cases was not possible, as there has not been enough information published about the reporter, consequences or clinical significance and the action taken following the recall or withdrawal. Additionally, the prevalence of quality failure of medicines in Sri Lanka could not be determined because the availability of the total number of medicines during that particular period is unknown, which is another limitation. We would also acknowledge that only a few defects have been published on the website in 2018, as the website was launched in the same year, and there could have been delays in publishing medicine alerts from the same year, which is a clear limitation.

## Conclusions

Substandard medicines are a problem in Sri Lanka similar to that of other LMICs. This study revealed that contamination was the most frequent cause of defective medicines, while parenteral preparations were the most susceptible pharmaceutical dosage form. Moreover, this study shows that some manufacturers were accountable for repetitive withholds and recalls, and these findings demonstrate the need for a control capacity in regulatory agencies and legislation that can impose relevant sanctions when necessary.

### Supplementary Information

**Additional file 1. **The defects by categories and subcategories with examples.

**Additional file 2. **Total number of withholds and recalls by ATC category.

**Additional file 3. **Total number of withholds and recalls of medicines according to pharmaceutical dosage forms.

## Data Availability

The datasets obtained and/or analysed during the current study are available from the corresponding author on reasonable request.

## Abbreviations

API: Active Pharmaceutical Ingredient
ATC: Anatomical Therapeutic Chemical
ATC/DDD: Anatomical Therapeutic Chemical/Defined Daily Dose
ADR: Adverse Drug Reaction
EMA: European Medicines Agency
FDA: Food and Drug Administration
FM: Falsified Medicines
GMP: Good Manufacturing Practices
LMIC: Low- or Middle-Income Countries
NMRA: National Medicines Regulatory Authority
NMQAL: National Medicines Quality Assurance Laboratory
SM: Substandard Medicines
USA: United States of America
UK: United Kingdom
WHO: World Health Organization
