# Supplementary material for: Quality of medicines in Sri Lanka: a retrospective review of safety alerts
Source: BMC Health Serv Res. 2023 Sep 12;23:980. doi: 10.1186/s12913-023-09995-3 (PMC10496228; doi:10.1186/s12913-023-09995-3)
Supplement: Supplementary file 1 — Additional file 1. The defects by categories and subcategories with examples. [file 12913_2023_9995_MOESM1_ESM.docx]

**Additional file 1** The defects by categories and subcategories with examples

| **Defect category** | **Total numbers N (%)** | **Subcategory** | **Total numbers (N)** | **Examples** |
| --- | --- | --- | --- | --- |
| 1.Contamination | 59 (36.2) | Impurities, related substances or other undeclared substances | 49 | - Colour variation   Presence of brown/black/yellow patches or spots on the surface of the medicines   - Presence of foreign matters Observation of a piece of glass/presence of yellow coloured dust/presence of floating particles - Out of specification of particulate/related substances   Sample does not conform to prescribed standard or specification with respect to the particulate matter test |
|  |  | Lack of sterility | 7 | Failure to comply with the test for sterility |
|  |  | Microbial contamination | 3 | Failure to comply with the test for microbial limits |
| 2. Stability defects | 41 (25.2) | Unspecified stability failure | 33 | - Physical stability defect   Tablets are easily broken into pieces when removing from the primary pack and mottled appearance of tablets. Melted capsules in the intact blister pack.   - Out of specification   Failure to comply with the test for friability. |
|  |  | Failure in dissolution test | 8 | Sample does not conform to standard specification with respect to the dissolution test.  Observation of orange-colored clumps in the content of some intact bottles. |
| 3. Active pharmaceutical ingredient (API) defects | 26 (15.9) | API out of specification (either more or less) | 26 | Sample does not conform to standard specification with respect to the test for uniformity of dosage unit and assay. |
| 4. Variation in physical properties such as weight and volume | 4 (2.5) | Weight variation | 3 | Failure to comply with the test for uniformity of weight. |
|  |  | Volume variation in single dose parenteral preparation | 1 | Sample does not conform to standard specification with respect to the extractable volume and observation of an ampoule as almost empty. |
| 5. Packaging and labelling defects | 27 (16.6) | Failure in container closure system functionality | 6 | Observation of leakage through the weak points of the seams which may have not sealed properly during the sealing process, or the damages may be due to improper packaging.  Leakage of the content through the lid of intact bottles. |
|  |  | Packaging in a wrong carton | 1 | Stating the strength of the medicine in two different ways on the same label, Primary label stated as 'Noradrenaline injection BP 2mg/2ml’. However, secondary label (outside the carton) stated as ‘each 2ml contains: Noradrenaline Acid Tartrate BP 4mg’ in the same product package. |
|  |  | The manufacturer's information missing | 4 | Missing the name and address of the manufacturer on the label. |
|  |  | Contain a lesser number of tablets than stated | 1 | Some boxes/sealed packs contain lesser number of tablets than stated on the label of the outer carton. |
|  |  | Failure to comply with the description as per the manufacturer’s specifications | 7 | Sample does not conform to the manufacturers’ specifications with respect to the description of the product. |
|  |  | Labelling errors | 8 | Discrepancy in labeling between commercial samples and specimen labels in the registration dossier.  Presence of a different label in addition to the respective primary label. |
| 6. Adverse events reported | 1 (0.6) | A cluster of adverse events adverse drug reaction (ADR) reported | 1 | Reports of cluster of adverse events (severe headache, soon after injection). |
| 7. Unclassified | 5 (3.0) | Data not adequate to assess | 5 | Sample does not conform to the BP specification (specification not stated).  Manufacturer/Marketing Authorization holder failed to respond to NMRA query on suitability of the product for pediatric dosing. |
| **Total number of defects reported** | **163 (100.0)** |  | **163** |  |
