# Supplementary material for: Quality of medicines in Sri Lanka: a retrospective review of safety alerts
Source: BMC Health Serv Res. 2023 Sep 12;23:980. doi: 10.1186/s12913-023-09995-3 (PMC10496228; doi:10.1186/s12913-023-09995-3)
Supplement: Supplementary file 2 — Additional file 2. Total number of withholds and recalls by ATC category. [file 12913_2023_9995_MOESM2_ESM.docx]

**Additional file 2** Total number of withholds and recalls by ATC category

| All medicines (n=143) | | | | | | | | | |
| --- | --- | --- | --- | --- | --- | --- | --- | --- | --- |
| All defects (n=163)* | | | | | | | | | |
|  | **Recalls** | | | | **Withholds** | | | | **Total** |
|  | Oral | Parenteral | Other | **Total** | Oral | Parenteral | Other | **Total** |  |
| **ATC group** |  |  |  |  |  |  |  |  |  |
| A - Alimentary tract and metabolism | 05 | 05 | 01 | **11** | 04 | 03 | - | **07** | **18** |
| B - Blood and blood forming organs | 02 | - | - | **02** | - | - | - | **-** | **02** |
| C - Cardiovascular system | 03 | 08 |  | **11** | 01 | 02 |  | **03** | **14** |
| D - Dermatologicals | - | - | 03 | **03** | - | - | - | **-** | **03** |
| G - Genito-urinary system and sex hormones | - | - | - | **-** | - | - | - | **-** | **-** |
| H - Systemic hormonal preparations, excluding sex hormones and insulins | 02 | - | - | **02** | 02 | - | - | **02** | **04** |
| J - Anti-infectives for systemic use | 11 | 20 | 05 | **36** | 10 | 13 | 01 | **24** | **60** |
| L - Antineoplastic and immunomodulating agents | 01 | 01 | - | **02** | 02 | 01 | - | **03** | **05** |
| M - Musculo-skeletal system | - | 01 | - | **01** | - | - | - | **-** | **01** |
| N - Nervous system | 12 | 02 |  | **14** | 09 | 02 | 02 | **13** | **27** |
| P - Antiparasitic products, insecticides and repellents | - | - | - | **-** | - | - | - | **-** | **-** |
| R - Respiratory system | - | - | 01 | **01** | - | - | 01 | **01** | **02** |
| S - Sensory organs | - | - | - | **-** | - | - | - | **-** | **-** |
| V - Various | - | 02 | 02 | **04** | - | 03 | - | **03** | **07** |
| **Total** | **36** | **39** | **12** | **87** | **28** | **24** | **04** | **56** | **143** |

*Some had more than one defect, refer Figure 1
