# Supplementary material for: Quality of medicines in Sri Lanka: a retrospective review of safety alerts
Source: BMC Health Serv Res. 2023 Sep 12;23:980. doi: 10.1186/s12913-023-09995-3 (PMC10496228; doi:10.1186/s12913-023-09995-3)
Supplement: Supplementary file 3 — Additional file 3. Total number of withholds and recalls of medicines according to pharmaceutical dosage forms. [file 12913_2023_9995_MOESM3_ESM.docx]

**Additional file 3** Total number of withholds and recalls of medicines according to pharmaceutical dosage forms

| **Category** | **Types of dosage forms** | **Total number (%)** |
| --- | --- | --- |
| Oral preparations | Tablets | 49 (34.2) |
|  | Capsules | 14 (9.8) |
|  | Oral solutions | 08 (5.6) |
| Parenteral preparations |  | 63 (44.0) |
| External preparations | Ophthalmic solutions | 02 (1.4) |
|  | Ear drops | 01 (0.7) |
|  | Topical preparations  Nebulizer | 05 (3.5)  01 (0.7) |
| **Total** |  | 1. **(100.0)** |
